# Changes in denture retention with denture adhesives and oral moisturizers for the oral cavity: an in vitro study

**DOI:** 10.1186/s12903-021-01800-z

**Published:** 2021-09-08

**Authors:** Naoya Ikemura, Yuji Sato, Junichi Furuya, Osamu Shimodaira, Kana Takeda, Takuya Kakuta, Kunihito Yamane, Noboru Kitagawa

**Affiliations:** Department of Geriatric Dentistry, Showa University School of Dentistry, Tokyo, Japan

**Keywords:** Adhesives, Dental laboratories, Dentures, Denture retention, Meals, Oral health

## Abstract

**Background:**

It is difficult to maintain complete dentures during meals in place. This in vitro study aimed to assess changes in denture retention between rest and function using denture adhesives and oral moisturizers in an oral cavity model.

**Methods:**

The following test samples were applied between the palatal plate and the edentulous jaw ridge model: denture adhesive, denture adhesive for dry mouth, oral moisturizer, and denture moisturizer. The retentive force was measured under two conditions: at rest while immersed in water and during function with a 2.5-kg load applied. The plate was pulled perpendicular to the occlusal plane and the retentive force was measured using a digital force gauge.

**Results:**

Under dry conditions, denture adhesive for dry mouth and oral moisturizer had a significantly higher retentive force than denture adhesive and denture moisturizer. After 30 min of immersion in water, the retentive force of the denture adhesive increased while that of the oral moisturizer decreased. After 30 min of function, the retentive force of the denture adhesive and denture adhesive for dry mouth remained high, while that of the oral moisturizer and denture moisturizer significantly decreased. Between rest and function, the retentive force of the denture adhesive and denture adhesive for dry mouth was high, and that of the oral moisturizer was low.

**Conclusions:**

Immediately after use, denture adhesive for dry mouth exhibited high retentive force, but retention gradually decreased due to its water content.

## Background

In Japan, the elderly population aged 65 years and older is increasing at an unprecedented rate, accounting for 28.4% of the total population (2019: Ministry of Internal Affairs and Communications). This is the highest number in history and is expected to further increase for the next 30 years [1]. This super-aging population is also important in dentistry. The number of remaining teeth increases as dental treatment progresses. Due to an increased life span, the number of patients requiring complete dentures is increasing, and complete prosthodontic treatment is expected to become difficult for various reasons. In particular, as aging progresses, it is often difficult to maintain complete dentures due to systemic and oral factors, such as multiple systemic diseases [2, 3], oral movement disorders caused by diseases [4, 5], progression of dry mouth caused by side effects of drugs [6, 7], and ridge resorption and mandibular changes caused by aging [8, 9]. For elderly patients with an aggravated edentulous jaw, denture adhesives are recommended for denture retention and stability during function [10]. However, denture adhesives are difficult to remove from the oral mucosa after use. Furthermore, residual denture adhesives are likely to become a breeding ground for oral bacteria, causing aspiration pneumonia [11, 12]. Dry mouth, which is common among elderly people, is expected to exacerbate the disadvantages of using denture adhesives. Therefore, for some cases, instead of denture adhesives, denture adhesives for dry mouth or oral moisturizers, which have good cleanability and moisturizing properties are recommended [13–15]. Ohno et al. [16] measured the retentive force of test samples after drying the plate and discovered that denture adhesives for dry mouth and oral moisturizers had a significantly higher retentive force than denture adhesives.

In the preliminary experiment, the retentive force of the palatal plate was measured under two conditions: dried and soaked in water for 1 min. The results suggested that denture adhesives for dry mouth and oral moisturizer could have a higher short-term retentive force than denture adhesives. However, clinically, the retentive force of dentures must be at least 30 min for eating. Therefore, long-term studies are necessary to further clarify the retentive force of dentures. There have been no reports on changes in denture retention over time with denture adhesives, denture stabilizers for dry mouth, and oral moisturizers. Therefore, the purpose of this study was to recreate the oral cavity during rest and function on a model and elucidate the changes in denture retention over time.

## Methods

### Test samples

Following a previous study [13], in this study, the test sample was placed between a model of the edentulous jaw ridge and the denture, and the retentive force of the denture was measured. The following four test samples were assessed: cream-type denture adhesive (NP; New Poligrip® Sa; Glaxo Smith Kline, Tokyo, Japan), gel-type denture adhesive for dry mouth (DM; Pitatto Kaiteki Gel®; NISHIKA), gel-type oral moisturizer (BT; Biotene Oral balance Jell®; T&K, Tokyo, Japan), and cream-type denture moisturizer (DW; Denture Wet®; DENTCARE).

#### Characteristics of the test samples

NP contains Na/Ca/methoxyethylene maleic anhydride copolymer salt. It contains white vaseline as a moisturizing ingredient and exhibits strong adhesive properties when moistened. DM contains sodium polyacrylate, a sticky substance, and sodium hyaluronate, a water-retaining ingredient. BT contains hydroxyethyl cellulose, an adhesive, and glycerin, a moisturizing agent. DW contains dextrin palmitate, a sticky substance, and squalane, a moisturizing ingredient.

### Palatal plate manufacturing

**Fig. 1:**
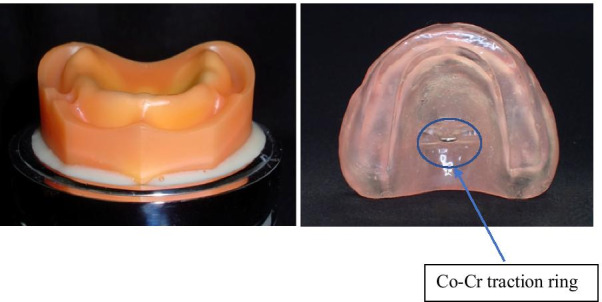
Silicone edentulous ridge model and articulating palatal plate. Impressions were made from a silicone edentulous ridge model. An articulating palatal plate was made and a 0.9 mm diameter Co-Cr traction ring was added to the center of the palate

Impressions of a silicone edentulous ridge model (G10FE-402K: Nissin Seiki) were made using silicone impression materials (Examixfine® Putty type and Injection type; GC, Tokyo, Japan), and an articulating palatal plate was fabricated using heat-cured resin (ACRON®; GC, Tokyo, Japan) to create a denture model. Regular relining (Tokuyama Rebase II; Tokuyama Dental) was performed in order to correct the polymerization shrinkage of the palatal plate. Further, a 0.9-mm traction ring made of Co-Cr was attached to the center of the palate (Fig. 1).

### Retentive force measuring device

**Fig. 2:**
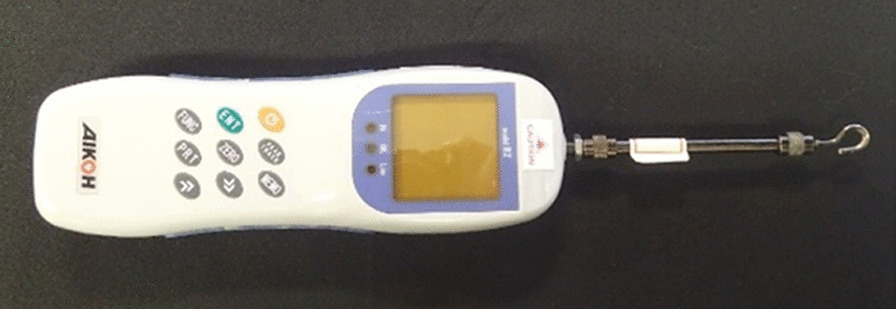
Digital force gauge. It is used to measure the retentive force along with a hook-shaped traction device that is used to pull palatal palate vertically

A digital force gauge (Digital Force gauge RX Series®; AIKOHENGINEERING, Tokyo, Japan) was used to measure the retentive force, with a hook-shaped traction device attached (Fig. 2).

### Measurement of retentive force in palatal plate

Approximately 2.5 g of the test sample was applied between the palatal plate and the model, such that the sample would cover the entire area by pressure welding. Then the overflowing test sample was removed and the palatal plate was pressed against the model similar to previous studies [13]. The different measurements were all performed on the model in a single attempt. A vertical load was applied for 10 s using a 2.5-kg disk weight, and the model was then immersed in tap water. In the experiment, the room temperature was set to 27.0 °C. Following this, the model was removed from the water, a 2.5-kg load was applied, and the plate was pulled at a speed of 1.0 N/s to the direction perpendicular to the occlusal plane using a digital force gauge. The force at which the palatal plate detached from the model was measured as the retentive force. The palatal plate was returned to the model without washing, and the load was applied to the model again; the maintenance force was measured by traction. Four measurements were taken, and the second to fourth measurements were averaged. The series of measurements were repeated three times, and the average value was used as the representative value.

### Experimental conditions

The retentive force was measured under two conditions: at rest, while immersed in water; and during function, with load equivalent to the occlusal pressure applied. The experiment was conducted using the four test samples.

**Fig. 3:**
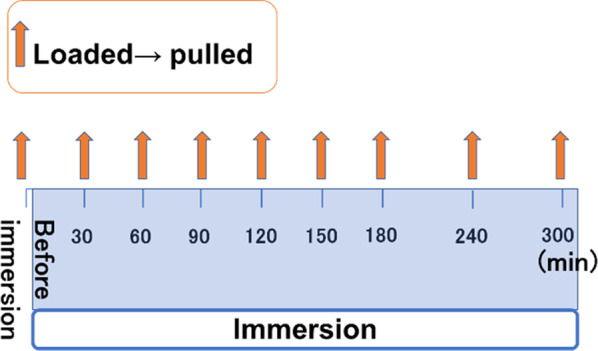
Resting retention experiment. Before immersion in water, a load was applied and the maintenance force was measured. The load was applied again and the specimens were immersed in water, and the maintenance force was measured every hour

**Fig. 4:**
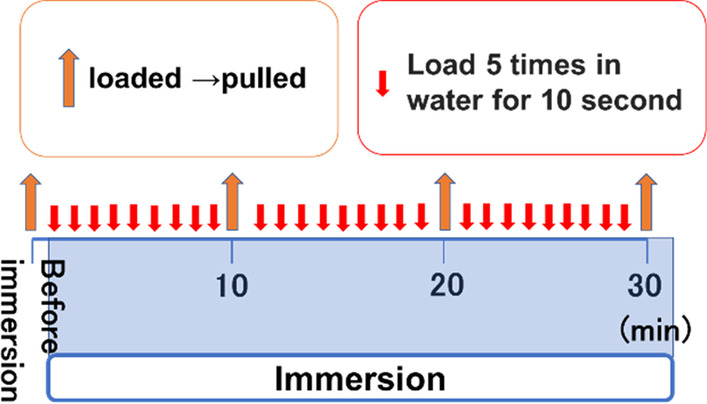
Experiment of retention during function. Before immersion in water, a load was applied and the maintenance force was measured. The load was applied again and the specimen was immersed in water and the load was applied in water. The specimens were removed from the water and the holding force was measured every hour

We applied 2.5 g of the test sample to the palatal plate, pressed against the model. The load was applied, the plate was pulled, and the retentive force was measured. After pressing the model again and applying a load, the model was immersed in water. After 30 min, it was removed from the water. The load was then applied, the plate was pulled, and the retentive force was measured. This was repeated every 30 min, and after 180 min, measurements were taken every 60 min. The retentive force at 300 min was considered as the change over time at rest (Fig. 3). Further, in order to measure the change over time during function, while submerged in water, a 2.5-kg load was applied five times in 10 s and the model was left for 20 s. Load was applied another five times for 10 s, and the model was left for 20 s. This process was repeated, and the model was removed from water every 10 min, and load traction was performed. The retentive force at 30 min was measured and was considered as the change over time during function. (Fig. 4).

### Statistical analysis

For statistical analysis, IBM SPSS Statistics 27.0 (Armonk, NY: IBM Corp) was used, one-way ANOVA was performed, and Tukey’s method was used for multiple comparisons. The significance level was set at *p* = 0.05.

## Results

### Changes over time in the retentive force at rest

**Fig. 5:**
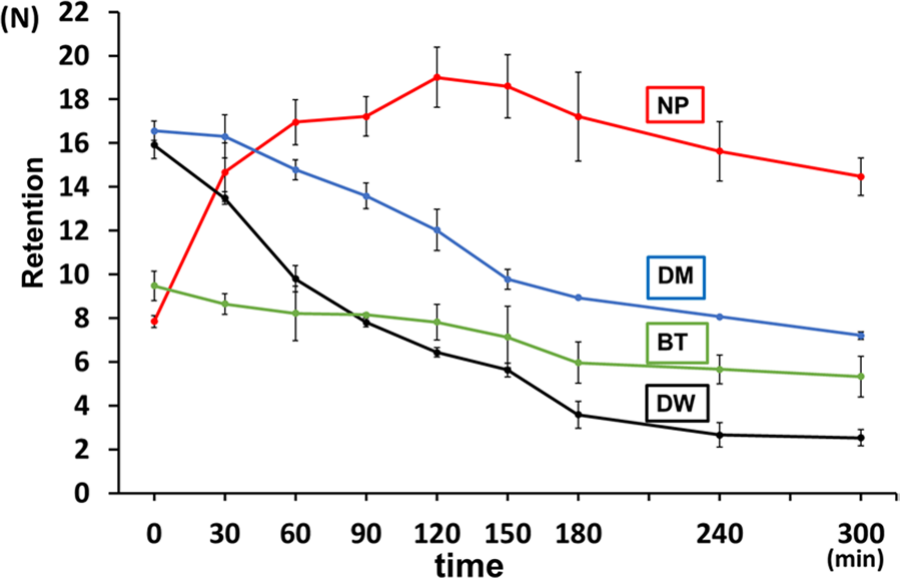
Change over time in the retentive force at rest. NP, New Poligrip® Sa; Glaxo Smith Kline, Tokyo, Japan (Cream-type denture adhesive); DM; Pitatto Kaiteki Gel®; NISHIKA (gel-type denture adhesive for dry mouth); BT, Biotene Oral balance Jell®; T&K, Tokyo, Japan (gel-type oral moisturizer); DW; Denture Wet®; DENTCARE (cream-type denture moisturizer)

Before immersion, the retentive force of DM and BT was significantly higher than that of NP and DW (*p* < 0.05). The significance level was set at *p* = 0.05. However, after 30 min of immersion in water, the retentive force of NP increased while that of BT decreased. After 60 min, the retentive force of NP and DM was significantly higher than that of BT and DW (*p* < 0.05). At 300 min, the retentive force of DM, BT, and DW decreased while that of NP remained high (Fig. 5). Table 1 shows the results of changes over time at rest.

**Table 1:** Results of changes over time in the retentive force at rest

	NP	DM	BT	DW
Before immersion	7.85	16.56	15.92	9.47
30 min	14.67	16.31	13.49	8.65
60 min	16.95	14.79	9.80	8.22
90 min	17.22	13.59	7.80	8.14
120 min	19.01	12.02	6.43	7.81
150 min	18.61	9.78	5.63	7.14
180 min	17.21	8.94	3.58	5.96
240 min	15.63	8.07	2.66	5.66
300 min	14.47	7.20	2.53	5.33

NP, New Poligrip® Sa; Glaxo Smith Kline, Tokyo, Japan (cream-type denture adhesive); DM; Pitatto Kaiteki Gel®; NISHIKA (gel-type denture adhesive for dry mouth); BT, Biotene Oral balance Jell®; T&K, Tokyo, Japan (gel-type oral moisturizer); DW; Denture Wet®; DENTCARE (cream-type denture moisturizer)

### Changes over time in the retentive force during function

**Fig. 6:**
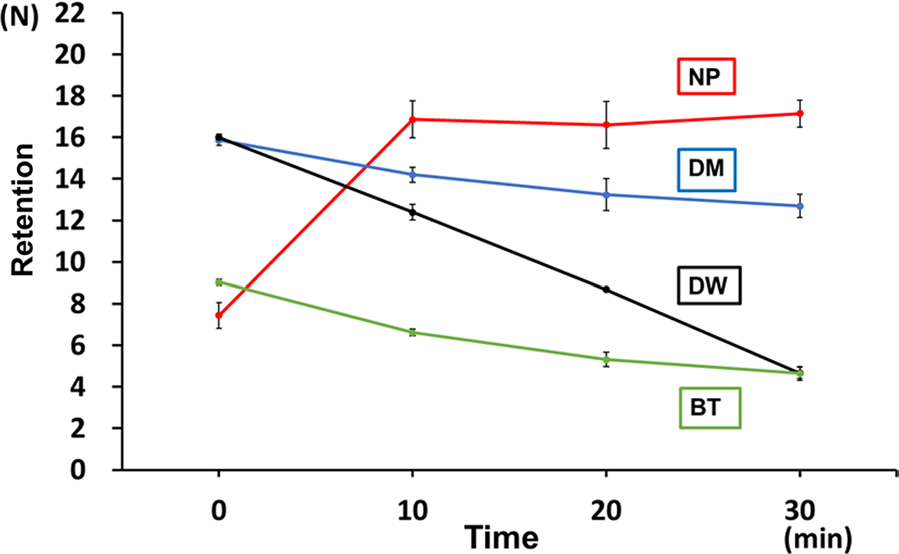
Changes over time in the retentive force during function. NP, New Poligrip® Sa; Glaxo Smith Kline, Tokyo, Japan (Cream-type denture adhesive); DM; Pitatto Kaiteki Gel®; NISHIKA (gel-type denture adhesive for dry mouth); BT, Biotene Oral balance Jell®; T&K, Tokyo, Japan (gel-type oral moisturizer); DW; Denture Wet®; DENTCARE (cream-type denture moisturizer)

During function, the retentive force of DM and BT before immersion was significantly lower than that of NP and DW (*p* < 0.05). However, after 10 min of immersion in water, the retentive force of NP significantly increased (*p* < 0.05) while that of BT rapidly decreased (*p* < 0.05). After 30 min, the retentive force of BT and DW significantly decreased. The retentive force of DM showed a slightly decreasing trend but remained high along with the retentive force of NP (Fig. 6). Table 2 below shows the changes over time in retentive force during function.

**Table 2:** Results of changes over time in the retentive force during function

	NP	DM	BT	DW
Before immersion	7.17	15.97	15.97	8.95
10 min	16.75	14.01	11.76	6.56
20 min	16.39	13.18	8.23	5.15
30 min	17.00	12.03	4.05	4.08

NP, New Poligrip® Sa; Glaxo Smith Kline, Tokyo, Japan (cream-type denture adhesive); DM; Pitatto Kaiteki Gel®; NISHIKA (gel-type denture adhesive for dry mouth); BT, Biotene Oral balance Jell®; T&K, Tokyo, Japan (gel-type oral moisturizer); DW; Denture Wet®; DENTCARE (cream-type denture moisturizer)

### Comparison of changes over time at rest and during function

**Fig. 7:**
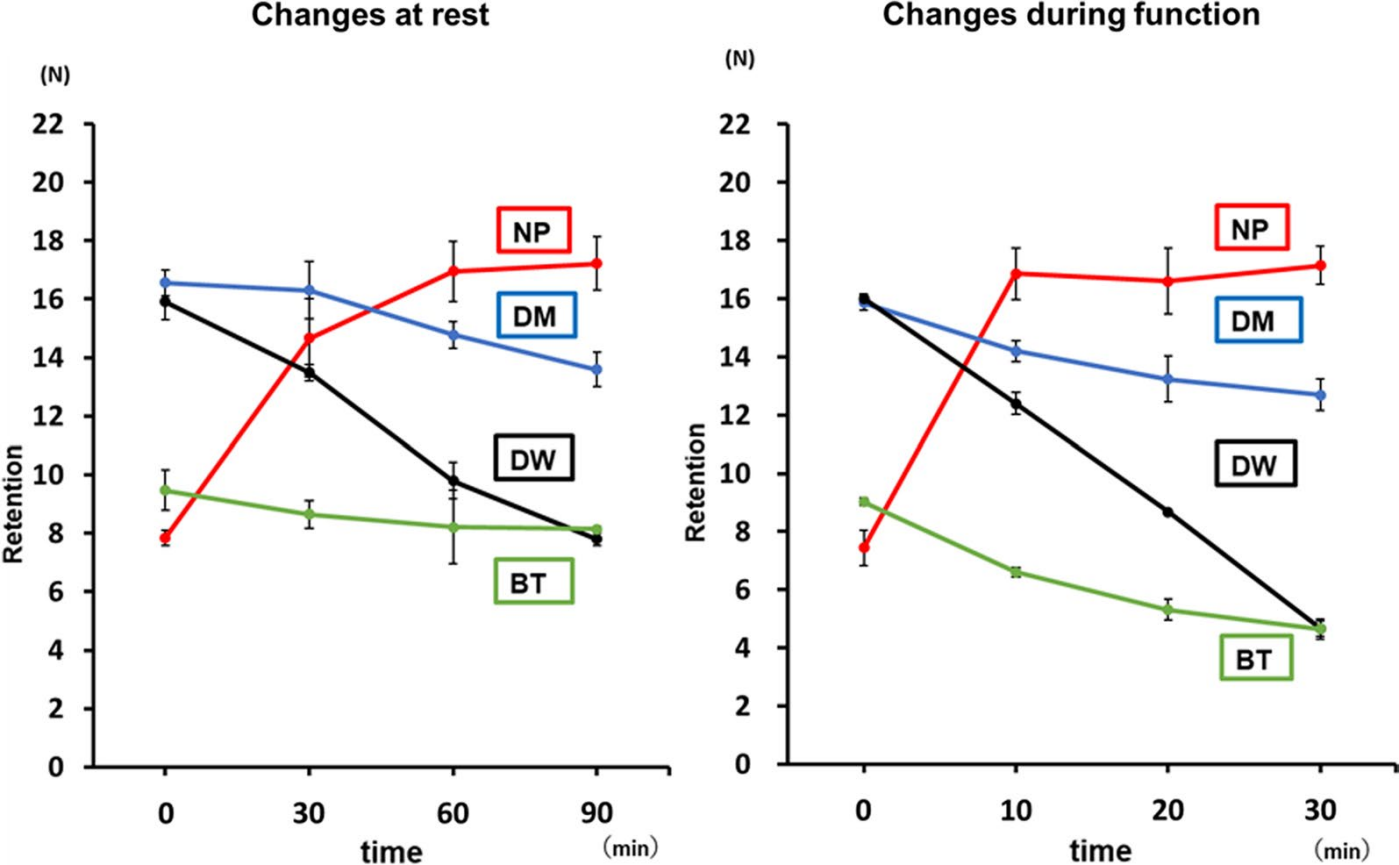
Comparison of changes over time at rest and during function. NP, New Poligrip® Sa; Glaxo Smith Kline, Tokyo, Japan (Cream-type denture adhesive); DM; Pitatto Kaiteki Gel®; NISHIKA (gel-type denture adhesive for dry mouth); BT, Biotene Oral balance Jell®; T&K, Tokyo, Japan (gel-type oral moisturizer); DW; Denture Wet®; DENTCARE (cream-type denture moisturizer)

When comparing the changes over time between rest and during function, the retentive force after 30 min during function showed the same tendency as that after 90 min of rest. The change over time in the retentive force during function was approximately three times higher than that at rest. The retentive force of NP and DM was high, and the retentive force of BT and DW was low (Fig. 7).

## Discussion

### Test samples

The amount of test sample placed between the model and the experimental bed was determined based on the results of preliminary experiments and past reports. In other words, when the entire surface of the model was sufficiently filled and the palatal plate was pressed, the amount of test sample overflowing from the periphery of the palatal plate was already determined. Yamagaki et al. [13] reported that when the entire model is filled with the test sample, the retentive force can be stably measured. In this study, stable measurements were achieved, similar to the preliminary experiment.

Furthermore, four types of test samples were assessed (NP, DM, highly viscous BT, and DW for different purposes), which can be used as alternatives with reference to previous studies.

### Changes over time in the retentive force at rest

Under dry conditions, DM and BT, which contain moisturizing ingredients, had a high retentive force. We believe that a certain degree of moisture is necessary to maintain the retentive force of dentures [17]. Although NP does not have moisturizing properties, it exerts its retentive force by containing water. Its retentive force increased with time immersed in water. Since BT is fluid, it flows into the water as the immersion time increases, causing its retentive power to decrease. However, since DM also has ingredients that exert retentive force by containing water, its retentive force was high to some extent [16]. Further, DW had a lower viscosity than other test samples, which may contribute to its low retentive force.

### Changes over time in the retentive force during function

When assessing the change in the retentive force over time, after 10 min, the increase in the retentive force of NP was the highest. The retentive force of BT decreased sharply. After 30 min, the retentive force of BT and DW was low, and the retentive force of NP slightly decreased. However, NP and DM had a high retentive force. We believe that the repeated application of the load caused a horizontal shift in the palatal plate, and the test sample between the palatal plate and the model was affected by water at an accelerated rate, causing a faster change over time in the retentive force.

### Comparison of changes over time at rest and during function

The change over time in the retentive force during function was approximately three times faster than that at rest. We believe that the repeated application of the load caused a horizontal shift in the palatal plate, and the test sample between the palatal plate and the model was affected by water at an accelerated rate, causing a sudden faster change over time in the retentive force during function. Assuming this occurs in the oral cavity, we believe that there is a considerable difference in the retentive force over time between rest and during eating and drinking.

Assuming an eating and drinking time of 60 min or longer, NP has a better retentive force. However, assuming general eating and drinking time, DM, which has good cleanability and moisturizing properties, can be useful for retaining dentures.

### Future outlook

In this study, we assumed dry mouth as the condition before immersion in water and healthy oral cavity as during immersion in water.

The major strength of this experiment was that since it was conducted using a model, we were able to investigate the changes in maintenance over time.

The drawback was that the water temperature and water viscosity were not set and were inadequate for comparison with the actual conditions in the oral cavity.

This study was able to compare the change in the maintenance force over time for each test sample at rest and at function, assuming an oral cavity. We wish to compare the results obtained on models with that of the oral cavity in a real patient in future in vivo studies. In an actual oral cavity, the ridge morphology and the elasticity and thickness of the mucosa differ, which affects the retentive force of the denture [15, 18]. Therefore, we would like to measure the retentive force of the denture in the oral cavity of patients with complete dentures and clarify the relationship between different conditions (at rest and during function) and the changes in retentive force over time. Further, we would like to clarify the relationship between the degree of dry mouth and the retentive force of dentures as well as the relationship between the objective retentive force evaluation and the satisfaction of subjects for each test sample.

### Clinical significance

The results of this study suggest that denture adhesives for dry mouth have some maintenance effect when examined over time at rest and during function. Compared to conventional denture stabilizers, the better cleaning properties of this product would help maintain good denture cleaning and oral cleaning conditions. This would lead to the prevention of aspiration pneumonia, oral candidiasis, and denture stomatitis, which are common problems among the elderly. In addition, patients with dry mouth are not familiar with conventional denture stabilizers, resulting in denture incompatibility. From the results of this study and that of Ohno et al. [16], denture adhesives for dry mouth can be applied immediately following use since they are familiar and retain moisture well. Conventional denture stabilizers are suitable for people with good cleaning and denture management skills; however, denture stabilizers for xerostomia are preferred for elderly people who have difficulty managing their own dentures due to frailty, sarcopenia, or dementia.

### Conclusions

Immediately after use, DM had high compatibility and exhibited retentive force, but retention gradually decreased due to its water content. On the contrary, immediately after use, the denture adhesive had low compatibility and exhibited low retentive force, but retention increased due to its water content. These findings suggest that denture adhesives for dry mouth, which have moisturizing properties, have a high retentive force for 30-min meals.

## Data Availability

The datasets used and/or analyzed during the current study are available from the corresponding author on reasonable request.

## Abbreviations

BT: Gel-type oral moisturizer
DM: Gel-type denture adhesive for dry mouth
DW: Cream-type denture moisturizer
NP: Cream-type denture adhesive
